# Comparing cancer incidence, stage at diagnosis and outcomes of First Nations and all other Manitobans: a retrospective analysis

**DOI:** 10.1186/s12885-019-6296-7

**Published:** 2019-11-06

**Authors:** Tara C. Horrill, Lindsey Dahl, Esther Sanderson, Garry Munro, Cindy Garson, Carole Taylor, Randy Fransoo, Genevieve Thompson, Catherine Cook, Janice Linton, Annette S. H. Schultz

**Affiliations:** 1 College of Nursing, Rady Faculty of Health Sciences, University of Manitoba, 89 Curry Place, Winnipeg, R3T 2N2 Canada; 2 Rady Faculty of Health Sciences, University of Manitoba, Winnipeg, Canada; 3 University College of the North, The Pas, Canada; 4 Cree Nation Tribal Health Centre, The Pas, Canada; 5 Interlake Reserves Tribal Council, Winnipeg, Canada; 6 Manitoba Centre for Health Policy, University of Manitoba, Winnipeg, Canada; 7 Indigenous Health Librarian, University of Manitoba, Winnipeg, Canada

**Keywords:** Cancer, Mortality, Neoplasms, Indigenous, First Nations, Canada

## Abstract

**Background:**

Globally, epidemiological evidence suggests cancer incidence and outcomes among Indigenous peoples are a growing concern. Although historically cancer among First Nations (FN) peoples in Canada was relatively unknown, recent epidemiological evidence reveals a widening of cancer related disparities. However evidence at the population level is limited. The aim of this study was to explore cancer incidence, stage at diagnosis, and outcomes among status FN peoples in comparison with all other Manitobans (AOM).

**Methods:**

All cancers diagnosed between April 1, 2004 and March 31, 2011 were linked with the Indian Registry System and five provincial healthcare databases to compare differences in characteristics, cancer incidence, and stage at diagnosis and mortality of the FN and AOM cohorts. Cox proportional hazard regression models were used to examine mortality.

**Results:**

The FN cohort was significantly younger, with higher comorbidities than AOM. A higher proportion of FN people were diagnosed with cancer at stages III (18.7% vs. 15.4%) and IV (22.4% vs. 19.9%). Cancer incidence was significantly lower in the FN cohort, however, there were no significant differences between the two cohorts after adjusting for age, sex, income and area of residence. No significant trends in cancer incidence were identified in either cohort over time. Mortality was generally higher in the FN cohort.

**Conclusions:**

Despite similar cancer incidence, FN peoples in Manitoba experience poorer survival. The underlying causes of these disparities are not yet understood, particularly in relation to the impact of colonization and other determinants of health.

## Comparing cancer incidence, stage at diagnosis and outcomes of first nations and all other Manitobans: a retrospective analysis

Globally, epidemiologic reporting of cancer incidence and outcomes among Indigenous peoples is a growing concern [1]. Historically, cancer among First Nations (FN) peoples in Canada was relatively unknown [2]; however, recent epidemiological evidence reveals increasing cancer incidence among FN peoples [3]. In addition, this evidence demonstrates that FN people are diagnosed at later stages of cancer, and their survival is poorer. While the emerging epidemiologic evidence is telling a story of growing concern, there remains significant gaps in data due to limitations in monitoring trends and reporting patterns [4].

## Background

In Canada, previous studies show an increase in cancer incidence in FN people compared to non-FN people, however this observed trend seems to be cancer site specific [5–10]. Cancer stage at diagnosis is an important prognostic indicator, and evidence suggests FN people are more likely to be diagnosed at later stages than non-FN Canadians [11–13]. Disparities in survival are equally concerning. Cancer mortality is higher among FN in Ontario than non-FN people [7], and while trends indicate the mortality for breast and colorectal cancers are decreasing among non-FN, they are increasing among FN people. Colorectal cancer mortality in particular increased 8-fold among FN people in Manitoba between 1984 and 2008 [11]. FN people experience significantly poorer cancer survival than non-FN in multiple Canadian provinces [5–7, 12, 14–16], independent of stage at diagnosis [14], income, or rural residency [16].

Similar disparities in cancer incidence and outcomes between Indigenous peoples and their non-Indigenous counterparts are reported elsewhere. In the United States, cancer mortality rates have progressively declined among non-Indigenous Americans, yet remain unchanged among Indigenous peoples [17–19]. Indigenous peoples in Australia are more likely to have advanced disease at diagnosis and less likely to receive certain cancer treatments [8, 20–22]. Similarly, Indigenous peoples in New Zealand (Maori) experience significantly higher incidence of cancer than non-Indigenous New Zealanders, with evidence of disparities in stage at diagnosis, treatments received, and survival [8, 23–25].

Emerging evidence demonstrates the shifting trends that are causing the widening disparity between FN people and the general population, yet epidemiological studies focused on cancer incidence and stage at diagnosis at the population level are sparse, particularly within the Canadian context. Within Manitoba, breast, colorectal and cervical cancers have been studied in the FN population, however, to date, no study has investigated general cancer trends. In this article, findings from a secondary analysis of provincial health administrative data are reported and address three objectives: a) to describe the demographics, comorbidities, site and stage of cancer at diagnosis among FN people and All Other Manitobans (AOM) who received a cancer diagnosis between April 1, 2004 and March 31, 2011; b) to compare annual crude and adjusted incidence rates for each cohort; c) to investigate mortality outcomes for each cohort. Supporting this research to address identified research gaps is an interdisciplinary team of researchers and FN community members.

## Methods

### Study context

In Canada, the term ‘Indigenous peoples’ is used to describe three distinct groups: First Nations (FN), Metis and Inuit. Among FN people, those registered with the federal government are referred to as “status First Nations” or “registered Indians”. As of 2016, Indigenous peoples represent approximately 4.9% of the Canadian population (36 million); of those, approximately 58.4% self-reported as FN [26]. Within Manitoba, Indigenous peoples represent approximately 18% of the population, of which 58.4% self-reported as FN, with nearly all (97.5%) identifying as status FN [27].

Healthcare services in Canada are publicly funded, providing universal coverage for all medically necessary hospital, physician, and specialist services. While often referred to as the “Canadian healthcare system”, delivery of healthcare services, including cancer control, is the responsibility of each provincial or territorial government, in essence creating a network of 13 healthcare systems. For status FN people living on reserve lands, healthcare services are delivered or funded by the federal government (public health, prevention and limited primary care), but hospital and physician services are provided by the provincial/territorial government regardless of status. Thus, provincial health administrative data contain information for all patients with a cancer diagnosis in Manitoba (FN and AOM).

### Study design and data sources

A population-based secondary analysis of administrative health services data of newly diagnosed adult cancer patients (≥ 18 years of age) within the province of Manitoba between April 1, 2004 and March 31, 2011 was conducted. Patients with a diagnosis of “non-melanoma skin & in situ skin” cancers were excluded. Multiple datasets housed in the Manitoba Population Research Data Repository (Repository) at the Manitoba Centre for Health Policy (MCHP) were linked in order to compare differences in socio-demographic and clinical characteristics, incidence trends over time, and health outcomes between status-FN and AOM. Data files in the repository do not contain names or other identifying information; an encrypted identifier allows linkage across files, while protecting privacy. The specific data files used in this study included:

1. The Manitoba Health Insurance Registry, which contains person-level demographic information and residential postal codes for nearly all Manitobans, including FN people;
2. Hospital Abstracts, which includes International Classification of Diseases (ICD-10-CA) diagnostic codes and Canadian Classification for Health Interventions (CCI) procedure codes for all hospital admissions in Manitoba;
3. Medical Services, which contains information on physician and nurse practitioner services provided in Manitoba (and the associated ICD-9-CM);
4. The Vital Statistics Mortality Registry, which contains records of each person who has died in Manitoba and the primary cause of death;
5. The Manitoba Cancer Registry (MCR), which contains records on all incident cases of diagnosed cancer, including cancer treatment, tumor characteristics, and cancer site and stage at diagnosis;
6. The Census of Canada aggregate data file, which contains information used to create quintiles of area-level income, a commonly used indicator of socioeconomic status; and
7. The Indian Registry System (IRS), a national database maintained by the Department of Indigenous Services Canada, lists all registered FN people to determine eligibility for benefits provided by the federal government. Identification of FN patients within administrative data requires linkage of the IRS with encrypted personal health numbers.

Approval for this study was obtained from the University of Manitoba Education & Nursing Research Ethics Board, the Manitoba Health Information Privacy Committee, CancerCare Manitoba, and the Health Information Research Governance Committee at Nanaandawewigamig (First Nations Health and Social Secretariat of Manitoba).

### Defining variables and statistical analyses for each objective

#### Objective 1: Cancer patient characteristics

Descriptive characteristics of FN and AOM patients with a diagnosis of cancer between 2004 and 2011 were measured at the time of diagnosis, and included: age, sex, area of residence, area-level income, and Charlson Comorbidity Index score. Residence was measured at the Regional Health Authority (RHA) level. Five RHAs in Manitoba are responsible for delivering health services within their designated geographic area, and the RHA corresponding to the patients’ postal code was used to indicate residence. Income quintiles, a predictor of health and health service use [26, 27], were calculated separately for urban (Winnipeg and Brandon) and rural (all other areas of Manitoba) areas based on the average household incomes for each Census dissemination area. Each patient was assigned the income quintile of the dissemination area that contained their postal code. The Charlson Comorbidity Index provided a valid measure of each patient’s health status at the time of first cancer diagnosis [28]. Comorbidities were identified using ICD-10-CA codes from the hospital discharge abstract and ICD-9-CM codes from the medical claims database during the one-year period prior to cancer diagnosis. Cancer stage is contained in the MCR according to the American Joint Committee on Cancer Staging system [29]. This system is used to stage the severity of cancer between stages I (least severe) and IV (most severe) based on the pathological and clinical characteristics of the cancer. A fifth ‘unknown’ category was used for cancers that could not be assessed. Finally, the site of cancer according to the International Classification of Diseases for Oncology Third Edition (ICD-O3) is also recorded in the MCR. Differences in these variables between FN and AOM cohorts were tested for significance using t-tests for continuous variables, and chi-squared tests for categorical variables.

#### Objective 2: Cancer incidence

Annual crude and adjusted rates of cancer incidence were calculated in the FN and AOM populations for each year within the study time period. For each annual rate, a count of all cancers diagnosed from the sixteen ICD-O3 site categories considered for this study were used as the numerators and the annual FN and AOM population counts of adults aged 18 years of age or older were used as the denominators. A generalized linear model with a negative binomial log link function was used to calculate adjusted annual incidence rates, controlling for age, sex, income quintile and area of residence recorded at the Regional Health Authority (RHA) level. Differences in incidence rates between FN and AOM populations were tested for significance using a chi-squared test and the trends over time were analyzed with linear regression models fit to the annual rates.

#### Objective 3: mortality

All-cause mortality and cancer-specific mortality were calculated for FN and AOM populations. Patients were followed for five years from the date of cancer diagnosis. Mortality information, including the date and primary cause of death, were identified using the Vital Statistics data file. Unadjusted and adjusted Cox proportional hazard regression models were used to examine the association between FN status and five year all-cause and cancer-specific mortality. Patient event times were calculated from the date of cancer diagnosis to the date of death, or were censored at five years if no evidence of death, or on the date of health insurance coverage discontinuation, which often indicates that the person has moved out of Manitoba. To examine the association between FN status and overall mortality, patient data was censored at the time of death for all non-cancer-related causes of death. When assessing cancer-specific mortality, a patient may die due to causes unrelated to the disease, therefore it was necessary to account for these competing risk events. Each adjusted model controlled for age, sex, RHA of residence, area-level income, Charlson comorbidity index, and the stage of cancer at time of diagnosis. All effect estimates are reported as hazard ratios with 95% confidence intervals and the significance level was *p* < 0.05. Analysis for each objective was done on the secure server at MCHP, using SAS statistical analysis software, V.9.4 (SAS Institute).

## Results

### Characteristics of patients with a first diagnosis of Cancer

In total, 38,076 adult Manitobans were diagnosed with a first cancer between 2004 and 2011, of which 1524 (4%) were FN people. FN people diagnosed with cancer were significantly younger than AOM (mean age 59.4 vs. 67.4 years; *p* < 0.0001). FN people diagnosed with cancer experienced significantly more co-morbidities as indicated by the Charlson Co-morbidity Index scores (1.4 vs. 1.0; p < 0.0001). There were higher proportions of FN people with a first time cancer diagnosis living in the lowest urban and rural income quintiles compared to AOM, with a decrease in proportion seen with increasing income. Among the AOM cohort, the proportions of patients within the urban and rural income quintiles were more evenly distributed (Table 1).

**Table 1:** Characteristics of Cancer Patients by FN Status and AOM

Characteristic	First Nations *n* = 1524 (4%)	AOM *n* = 36,552 (96%)	*p* value
Age (years) mean ± SD	59.4 ± 14.4	67.4 ± 14.1	< 0.0001
Female Sex	845 (55.4%)	18,386 (50.3%)	< 0.0001
Regional Health Authority			< 0.0001
Interlake-Eastern	338 (22.2%)	3838 (10.5%)	
Northern	535 (35.1%)	724 (2.0%)	
Southern Health	88 (5.8%)	4213 (11.5%)	
Prairie Mountain Health	168 (11.0%)	5873 (16.1%)	
Winnipeg	382 (25.1%)	21,728 (59.4%)	
Public Trustee	13 (0.9%)	176 (0.5%)	
Average household income quintiles			< 0.0001
Rural 1 (lowest rural)	587 (38.5%)	1876 (5.1%)	
Rural 2	252 (16.5%)	2957 (8.1%)	
Rural 3	86 (5.6%)	3009 (8.2%)	
Rural 4	126 (8.3%)	2755 (7.5%)	
Rural 5 (highest urban)	61 (4.0%)	2364 (6.5%)	
Urban 1 (lowest urban)	219 (14.4%)	4963 (13.6%)	
Urban 2	79 (5.2%)	4896 (13.4%)	
Urban 3	42 (2.8%)	4815 (13.2%)	
Urban 4	36 (2.4%)	4310 (11.8%)	
Urban 5 (highest urban)	15 (1.0%)	4103 (11.2%)	
Charlson Comorbidity Index Score, mean ± SD	1.4 ± 1.3	1.0 ± 1.1	< 0.0001
Myocardial infarction	46 (3%)	704 (1.9%)	
Congestive heart failure	106 (7.0%)	2200 (6.0%)	
Peripheral vascular disease	53 (3.5%)	1175 (3.2%)	
Cerebrovascular disease	62 (4.1%)	1469 (4.0%)	
Dementia	21 (1.4%)	1146 (3.1%)	
Chronic pulmonary disease	263 (17.3%)	5544 (15.2%)	
Connective tissue disease	56 (3.7%)	726 (2.0%)	
Peptic ulcer disease	73 (4.8%)	602 (1.6%)	
Mild liver disease	66 (4.3%)	678 (1.9%)	
Diabetes without complications	514 (33.7%)	5286 (14.5%)	
Diabetes with complications	105 (6.9%)	656 (1.8%)	
Paraplegia and hemiplegia	14 (0.9%)	214 (0.6%)	
Renal disease	83 (5.4%)	962 (2.6%)	
Cancer	519 (34.1%)	13,515 (37.0%)	
Moderate or severe liver disease	14 (0.9%)	153 (0.4%)	
Metastatic carcinoma	71 (4.7%)	1059 (2.9%)	
HIV/AIDS	8 (0.5%)	20 (0.1%)	

### Cancer diagnoses by stage & site

Statistically significant differences in cancer stage at diagnosis were seen between the FN and AOM cohorts, with a higher proportion of FN patients diagnosed at stages III and IV (Table 2). Cancer site analysis also demonstrated statistically significant differences between FN patients and AOM. Notably, a significantly higher proportion of FN patients were diagnosed with cervical cancer, kidney cancer, and colorectal cancer (Table 3). A significantly lower proportion of FN patients were diagnosed with prostate cancer, melanoma, chronic lymphocytic leukemia, and bladder cancer. Given small numbers of some cancers, we are not able to report incidence of cancers by site and sex.

**Table 2:** Cancer Stage at Diagnosis by FN Status and AOM

Cancer Stage	First Nation (*n* = 1524)	AOM (*n* = 36,552)	*p* Value
I	362 (23.8%)	9217 (25.2%)	0.1972
II	340 (22.3%)	8763 (24.0%)	0.1356
III	285 (18.7%)	5637 (15.4%)	0.0005
IV	342 (22.4%)	7277 (19.9%)	0.0155
Unknown	195 (12.8%)	5658 (15.5%)	0.0044

**Table 3:** Cancer Site by FN Status

Cancer Site	FN (*n* = 1524)	AOM (*n* = 36,552)	*p* value
Bladder	13 (0.9%)	814 (2.2%)	0.0003
Breast	210 (13.8%)	5219 (14.3%)	0.5853
Cervix	43 (3.0%)	286 (0.8%)	< 0.0001
Chronic Lymphocytic Leukemia	7 (0.5%)	351 (1.8%)	0.0001
Colorectal	240 (15.7%)	5063 (13.9%)	0.0362
Kidney	136 (8.9%)	1177 (3.2%)	< 0.0001
Lung & Bronchus	205 (13.5%)	5306 (14.5%)	0.247
Melanoma	8 (0.5%)	988 (2.7%)	< 0.0001
Non-Hodgkin Lymphoma	65 (4.6%)	1609 (4.4%)	0.7985
Ovarian	32 (2.1%)	545 (1.5%)	0.0567
Pancreas	32 (2.1%)	914 (2.5%)	0.3247
Prostate	134 (8.8%)	4558 (12.5%)	< 0.0001
Stomach	29 (1.9%)	727 (2.0%)	0.8135
Thyroid	20 (1.3%)	667 (1.8%)	0.1409
Uterine	38 (2.5%)	1263 (3.5%)	0.0428
Other	355 (23.3%)	7051 (19.3%)	0.0714

### Cancer Incidence & Trends

The overall crude cancer incidence rate between 2004 and 2011 was significantly lower in the FN population (334.90 vs. 651.57 per 100,000; *p* < 0.0001), and annual crude cancer incidence rates were lower in the FN population for each year within the study period (Table 4). However, after adjusting for age, sex (Table 5), and further adjusting for income and area of residence (Table 6), there were no differences in the annual incidence rates, except for the year 2008/09 in which FN patients had a higher incidence once adjusted for both age and sex, and income and area of residence (331.9 vs. 278.6 per 100,000; *p* = 0.0171) (Table 6). There were no significant trends over time in either cohort.

**Table 4:** Crude Rates of Cancer Incidence (total) per 100,000 by FN Status

Fiscal Year	First Nation			AOM			*p* value
	Count	IR per 100,000	95% CI	Count	IR per 100,000	95% CI	
2004/2005	191	306.04	264.09–350.96	5383	653.55	635.96–670.88	< 0.0001
2005/2006	194	303.15	264.81–350.63	5226	631.49	614.84–649.10	< 0.0001
2006/2007	202	307.90	268.24–353.43	5393	648.05	630.99–665.58	< 0.0001
2007/2008	209	310.90	271.48–356.04	5473	649.37	632.62–667.04	< 0.0001
2008/2009	273	395.75	351.48–445.59	5679	666.46	649.23–683.90	< 0.0001
2009/2010	256	359.43	317.99–406.27	5691	657.56	640.59–674.75	< 0.0001
2010/2011	259	352.24	311.85–397.86	5749	653.76	637.19–670.94	< 0.0001
Overall	1584	334.90	318.81–351.81	38,594	651.57	645.11–658.11	< 0.0001

**Table 5:** Annual incidence rates (adjusted for age and sex)

Fiscal Year	First Nation			AOM			*p*-value
	Count	IR per 100,000	95% CI	Count	IR per 100,000	95% CI	
2004/2005	191	265.68	212.00–332.94	5383	259.21	218.07–308.11	0.864
2005/2006	194	262.48	209.67–328.60	5226	259.54	218.31–308.57	0.938
2006/2007	202	263.65	210.93–329.55	5393	268.04	225.47–318.65	0.908
2007/2008	209	254.06	203.46–317.25	5473	265.58	223.44–315.67	0.756
2008/2009	273	332.64	261.32–398.34	5679	268.58	226.00–319.19	0.184
2009/2010	256	290.99	234.95–360.40	5691	265.23	223.14–315.26	0.505
2010/2011	259	283.98	229.42–351.51	5749	262.61	220.97–312.10	0.574
Overall	1584	272.21	228.18–324.73	38,594	265.20	224.40–313.43	0.832

**Table 6:** Annual incidence rates (adjusted for age, sex, income and area of residence)

Fiscal Year	First Nation			AOM			*p*-value
	Count	IR per 100,000	95% CI	Count	IR per 100,000	95% CI	
2004/2005	191	263.76	224.37–306.40	5383	281.01	265.35–296.73	0.449
2005/2006	194	261.44	225.45–306.74	5226	267.29	253.31–283.38	0.790
2006/2007	202	260.62	223.92–303.33	5393	276.97	261.36–292.26	0.457
2007/2008	209	259.80	223.70–301.73	5473	270.22	256.07–286.23	0.626
2008/2009	273	331.88	290.18–379.57	5679	278.60	263.34–294.27	0.017
2009/2010	256	301.92	263.45–346.00	5691	274.63	259.96–290.14	0.201
2010/2011	259	295.81	255.66–335.36	5749	273.99	259.37–289.49	0.369
Overall	1584	274.51	255.01–295.50	38,594	274.27	262.71–286.34	0.983

### Cancer mortality

The FN cohort had a significantly higher risk of all-cause mortality than the AOM cohort, both before (HR 1.12 95% CI 1.045–1.196) and after adjustment (HR 1.26, 95% CI 1.178–1.351), and significantly higher risk of all cause-mortality 5 years post cancer diagnosis (HR 1.23, 95% CI 1.152–1.321) (Table 7). The FN cohort also had a higher risk of *overall* cancer-specific mortality in both the crude (HR 1.126, 95% CI 1.046–1.211), and adjusted models (HR 1.108, 95% CI 1.009–1.218), and in *5-year* cancer-specific mortality (HR 1.099, 95% CI 1.001–1.207).

**Table 7:** Mortality Hazard Ratios^a^

	HR	95% CI	*P*-value
Crude All-Cause Mortality	1.119	1.045–1.196	0.0011
Adjusted All-Cause Mortality	1.262	1.178–1.351	< 0.0001
Adjusted All-Cause Mortality 5 Years Post-Diagnosis	1.234	1.152–1.321	< 0.0001
Crude Cancer-Specific Mortality	1.126	1.046–1.211	0.0015
Adjusted Cancer-Specific Mortality	1.108	1.009–1.218	0.0322
Adjusted Cancer-Specific Mortality 5 Years Post-Diagnosis	1.099	1.001–1.207	0.0487

^a^Adjusted models have controlled for age, sex, area of residence by RHA, area-level income, Charlson comorbidity index, and stage of cancer at time of diagnosis

## Discussion

This study aimed to describe the characteristics of FN patients and AOM diagnosed with cancer between 2004 and 2011, and examine cancer incidence, site, stage at diagnosis, and mortality. Our results indicate that the FN cohort was significantly younger, and had a significantly higher Charlson Comorbidity Index compared to the AOM cohort. Although crude incidence rates among the FN cohort were half that of AOM, these differences were not sustained after adjusting for age, sex, income and area of residence. We found no significant trends in cancer incidence in either cohort over time. We did, however, find significant differences in cancer sites diagnosed between cohorts. Notably, our results show a higher proportion of FN patients diagnosed with cancer at stages III and IV than AOM, and a higher risk of all-cause mortality and cancer-specific mortality in the FN cohort.

We found that the proportion of prostate, bladder, and uterine cancers were significantly lower in the FN cohort, while the proportion of cervical, colorectal and kidney cancers were significantly higher in the FN cohort compared to AOM. Elsewhere in Canada, incidence of bladder, breast and uterine cancers and melanoma were lower among FN people in Ontario [7], and lower incidence of breast and prostate cancers were found among FN people in British Columbia [6]. A significantly higher incidence of colorectal and cervical cancers have been found among FN people in British Columbia and Manitoba [6, 9, 11], and a significantly higher incidence of colorectal, kidney and cervical cancers among FN people in Ontario [5, 7]. Differences in cancer incidence may be related to genetic risk or environmental exposures, however, we wish to draw attention to alternative factors that may explain, in part, some of these differences. Higher proportions of cervical cancer among FN women may suggest poor access to screening services, which identify pre-cancerous changes that can be treated to prevent cancer. A recent meta-analysis found increased risk of invasive cervical cancer and cervical cancer-related mortality among Indigenous women compared to non-Indigenous women, yet no increased risk of cervical dysplasia or carcinoma in situ (precursors to cervical cancer) [30]. These results suggest “structural, social, or individual barriers to screening, rather than baseline risk factors, are influencing poor health outcomes” [30, p148].

Poor access to cervical cancer screening may be related to geographical availability of services as well as access to culturally safe services, which are particularly important within the context of historical trauma and experiences of residential school survivors, and the invasive nature of cervical cancer screening [31–34]. Within Canada, research indicates Indigenous women face multiple structural barriers to accessing cervical cancer screening (including historical, political, socioeconomic, and health systems factors), many of are rooted in colonial history [32, 33]. Within Manitoba, FN women over 40 are less likely to receive a pap test than AOM, FN women younger than 25 are more likely to receive a pap test, and there is no difference between FN and AOM in pap testing for women 25–39 [9]. Higher incidence of cervical cancer among FN women may also indicate poor access to follow-up care after an abnormal Pap test result [6, 33].

In our study, a higher proportion of FN people compared with AOM were diagnosed with cancer at stages III (18.7% vs. 15.4%) and IV (22.4% vs. 19.9%). Several other Canadian studies have demonstrated similar patterns of late-stage diagnosis, with FN women more likely to be diagnosed with breast cancer at later stages than non-FN women [11–13]. This is particularly concerning given that cancer stage at diagnosis has a significant impact on treatment options and cancer outcomes, and is an important indicator of the quality of, and access to screening and early detection services [35, 36]. FN people in Canada experience difficulty accessing primary care [37, 38] and diagnostic services [39, 40], which may be contributing to higher rates of stage III and IV diagnoses. Many FN communities are located in rural or remote areas characterized by low population density, poor transportation infrastructure, limited resources for diagnostics and high turnover of healthcare professionals. This results in limited or non-existent access to local healthcare services, poor continuity of care, and compromised quality of care [34, 40–44]. As such, many FN patients must travel to access basic diagnostic services, treatment and supportive care. Lengthy travel time, coupled with transportation that is not feasible, convenient or affordable creates significant barriers to accessing cancer care [45–47]. Although there are resources to support medical travel, particularly for status FN people, accessing these resources can come with challenges [39, 42].

Accessing health care, however, requires more than service or healthcare provider availability – patients must also feel that their concerns are heard, and that care will be provided that is free of judgment and culturally safe [48]. Cultural safety is an approach to delivering care that is based on establishing trusting and reciprocal relationships between a patient and their healthcare provider [49]. Lack of culturally safe care has been noted to be a barrier to accessing cancer care among FN people [34, 45, 50, 51]. Racism, discrimination, and fear of judgment have been noted to impede access to both primary care [37, 38] and cancer care [32, 47, 50, 52, 53], by causing patients to delay or avoid accessing care. These experiences are further exacerbated by histories of historical trauma, residential school attendance and Indian hospitals, which have been noted to increase distrust of healthcare providers. Feelings of distrust, negative experiences within institutional settings, culturally incongruous systems and experiences with marginalization and racism can result in patients delaying or avoiding seeking care [42, 46, 47, 53]. It is unclear to what extent healthcare providers are aware of the impact of their actions on FN peoples access to healthcare, and more research is needed to understand this relationship.

Finally, our results show that FN people had a higher risk of all-cause mortality and cancer-specific mortality than AOM both before and after adjustment for age, sex, cancer stage at diagnosis, income, region of residence and comorbidities. Our results also show that FN patients had a higher risk of death from *any cause* (HR 1.234, 95% CI 1.15–1.32, *p* < 0.0001) and a higher risk of *cancer-related* death (HR 1.099, 95% CI 1.001–1.207) at 5 years post-cancer diagnosis than AOM. These results are similar to other studies in Canada, indicating higher cancer-related mortality among FN people [6, 7, 14–16].

While disparities in cancer-related survival are multifactorial, the main determinant of survival is cancer stage at diagnosis [29]. Underlying these disparities, however, are a host of health inequities experienced by FN people in Canada, some of which are discussed above. Health inequities are the systematic and unjust differences in health between socioeconomic groups; these differences are generated by social, economic and environmental factors and contexts amenable to change, and are not a result of ‘lifestyle’ or personal choices [54]. Within Canada, a significant body of evidence demonstrates the substantial health inequities experienced by Indigenous peoples (including FN people) [55, 56]. Researchers, healthcare providers and policy makers must consider the context of these inequities, and how they are, at least in part, “the direct and indirect present-day symptoms of a history of loss of lands and autonomy and the results of the political, cultural, economic and social disenfranchisement that ensued” ([57], p59). Although individual characteristics, comorbidities, tumor biology, cancer treatment impact, and access to/use of healthcare services impact survival [6, 15, 58], an agenda to improve cancer outcomes among Indigenous peoples, including FN people, must also acknowledge and address health and social inequities. In particular, the tendency to focus on ‘lifestyle’ or behavioral risk factors (i.e., smoking, diet, alcohol) and education about risk factors, while ignoring the “drivers of these behaviors” must be disrupted ([59], pS517]). Our intent here is to draw attention to the ‘causes of the causes’ and determinants of Indigenous health, rather than perpetuate the discourse that focuses solely on genetic and ‘lifestyle’ risk factors as potential causes of the disparities and inequities described.

### Limitations

There are several limitations to this study. First, only those individuals registered under the *Indian Act* were included in the FN cohort, with non-registered FN people subsequently included with AOM. This may have resulted in an underrepresentation of the differences in stage at cancer diagnosis and outcomes given that non-registered FN people experience many of the same socioeconomic conditions, access to healthcare issues, and colonial history as registered FN people. At present, there is no mechanism to identify non-registered FN people in these datasets. Second, we were not able to analyze differences in mortality between the FN and AOM cohorts by disease site, and there may be significant differences in mortality and survival depending on cancer site. Further investigation is needed.

## Conclusion

Our study found no significant differences in overall adjusted cancer incidence between FN people and AOM, and no significant trends over time in overall cancer incidence in either cohort. However, a significantly higher proportion of FN people were diagnosed with cancer at stages III and IV compared to AOM. FN people also experienced higher all-cause mortality and cancer-specific mortality. No significant differences were seen between cohorts in 5-year site-specific mortality. The underlying causes of these disparities are complex, and not yet well understood, particularly in relation to the impact of colonization and other structural determinants of health. Further research is needed to better understand the complex and interactive nature of factors resulting in later cancer diagnoses among FN people.

## Data Availability

The data that support the findings of this study are available from the Manitoba Center for Health Policy, but restrictions apply to the availability of these data, which were used under license for the current study, and so are not publicly available.

## Abbreviations

AOM: All other Manitobans
FN: First Nations
ICD: International Classification of Diseases
MCHP: Manitoba Center for Health Policy
RHA: Regional health authority
